# Regulation of macrophage polarization by targeted metabolic reprogramming for the treatment of lupus nephritis

**DOI:** 10.1186/s10020-024-00866-z

**Published:** 2024-06-25

**Authors:** Limei Zhao, Shuqin Tang, Fahui Chen, Xiya Ren, Xiutao Han, Xiaoshuang Zhou

**Affiliations:** 1 The Fifth Clinical Medical College of Shanxi Medical University, Xinjian South Road No. 56, Yingze District, Taiyuan, Shanxi 030001 China; 2 The Third Clinical College, Shanxi University of Chinese Medicine, Jinzhong, Shanxi 030619 China; 3 Department of Nephrology, Shanxi Provincial People’s Hospital, The Fifth Clinical Medical College of Shanxi Medical University, Shuangta East Street No. 29, Yingze District, Taiyuan, Shanxi 030012 China

**Keywords:** Energy metabolism, Macrophages, Polarization, Lupus nephritis

## Abstract

Lupus nephritis (LN) is a severe and common manifestation of systemic lupus erythematosus (SLE) that is frequently identified with a poor prognosis. Macrophages play an important role in its pathogenesis. Different macrophage subtypes have different effects on lupus-affected kidneys. Based on their origin, macrophages can be divided into monocyte-derived macrophages (MoMacs) and tissue-resident macrophages (TrMacs). During nephritis, TrMacs develop a hybrid pro-inflammatory and anti-inflammatory functional phenotype, as they do not secrete arginase or nitric oxide (NO) when stimulated by cytokines. The infiltration of these mixed-phenotype macrophages is related to the continuous damage caused by immune complexes and exposure to circulating inflammatory mediators, which is an indication of the failure to resolve inflammation. On the other hand, MoMacs differentiate into M1 or M2 cells under cytokine stimulation. M1 macrophages are pro-inflammatory and secrete pro-inflammatory cytokines, while the M2 main phenotype is essentially anti-inflammatory and promotes tissue repair. Conversely, MoMacs undergo differentiation into M1 or M2 cells in response to cytokine stimulation. M1 macrophages are considered pro-inflammatory cells and secrete pro-inflammatory mediators, whereas the M2 main phenotype is primarily anti-inflammatory and promotes tissue repair. Moreover, based on cytokine expression, M2 macrophages can be further divided into M2a, M2b, and M2c phenotypes. M2a and M2c have anti-inflammatory effects and participate in tissue repair, while M2b cells have immunoregulatory and pro-inflammatory properties. Further, memory macrophages also have a role in the advancement of LN. Studies have demonstrated that the polarization of macrophages is controlled by multiple metabolic pathways, such as glycolysis, the pentose phosphate pathway, fatty acid oxidation, sphingolipid metabolism, the tricarboxylic acid cycle, and arginine metabolism. The changes in these metabolic pathways can be regulated by substances such as fish oil, polyenylphosphatidylcholine, taurine, fumaric acid, metformin, and salbutamol, which inhibit M1 polarization of macrophages and promote M2 polarization, thereby alleviating LN.

## Highlights

Macrophages are the main infiltrating cells in lupus nephritis (LN) kidneys. Different macrophage subtypes have different effects on the development and progression of LN. M1-type macrophages possess pro-inflammatory functions, participating in the onset and progression of LN. In contrast, M2-type macrophages primarily exhibit anti-inflammatory and tissue repair-promoting functions, which are beneficial for the remission of LN. The infiltration of mixed phenotype macrophages reflects a failure in the resolution of inflammation and correlates with poor prognostic outcomes in LN.

Multiple metabolic pathways, such as glycolysis, pentose phosphate pathway, amino sugar and nucleotide sugar metabolism, fatty acid oxidation, sphingolipid metabolism, tricarboxylic acid cycle, arginine metabolism, and tryptophan metabolism, regulate macrophage polarization.

Compounds such as omega-3 fatty acids, polyunsaturated phospholipids, taurine, fumarate, metformin, salbutamol, and others can modulate metabolic pathways, suppressing M1 polarization and promoting M2 polarization in macrophages, thereby ameliorating LN symptoms.

## Introduction

Lupus Nephritis (LN) is a prevalent and serious complication of systemic lupus erythematosus (SLE), affecting ≥ 88% of SLE patients throughout the disease condition. Among them, 44% will develop End-Stage Renal Disease (ESRD), a critical predictor of SLE mortality (Luan et al. 2022). The primary characteristic of LN is the accumulation of immunoglobulin G (IgG) in the kidneys, which leads to the activation of local immune cells, such as macrophages, and complement *via* interaction with Fcγ receptors (FcγRs) (Richoz et al. 2022).

Macrophages are a key component of the mononuclear phagocytic system of the kidney and play an important role in the defense against infection, kidney injury, and the repair of kidney damage (Bell and Conway 2022). Renal macrophages can be characterized based on the manifestation of pro-inflammatory and anti-inflammatory features, as well as their specific origins (Kwant et al. 2022). Their phenotype is influenced by signals from renal parenchymal cells. Injured renal cells may release danger-associated molecules that may stimulate the development of a pro-inflammatory phenotype. This phenotype can further recruit inflammatory cells, exacerbate renal injury, and activate renal fibroblasts to promote scar formation. The macrophages revert to their more reparative phenotype, inhibiting immune responses and promoting renal tissue repair, once the injurious stimulus has subsided (Bell and Conway 2022).

In LN patients, renal biopsies have revealed the presence of different macrophage subtypes in various kidney regions. The proportions of these subtypes are associated with the course of LN and histological categories (Kwant et al. 2022). Studies have demonstrated that metabolic reprogramming may regulate macrophage polarization in LN, thereby improving LN by modifying the proportions of the subtypes (Lee et al. 2011; Noe et al. 2021). Thus, it is of the utmost importance to comprehend and target the metabolic reprogramming of macrophages in patients with LN to modify the direction of polarization, thereby enhancing the prognosis of patients with SLE and preventing or delaying the progression of LN to ESRD.

## Role of different macrophage subtypes in LN

Macrophages play crucial roles in LN, with different macrophage subtypes exhibiting distinct pathogenic roles and functions. Based on their origin, they can be classified as either monocyte-derived macrophages (MoMacs) or tissue-resident macrophages (TrMacs). TrMacs are seeded from the yolk sac or fetal liver progenitor cells into organs before birth, after which they are replaced by different monocyte precursors (Liu et al. 2019). They comprise the highest population of renal macrophages, which accounts for approximately half of the total macrophage population. TrMacs are restricted to specific tissue compartments, including the renal tubulointerstium and glomeruli, where they express high levels of F4/80 and show limited circulation (Schulz et al. 2012). In LN, TrMacs express monocyte chemotactic factors but have limited uptake of immune complexes (Richoz et al. 2022). Studies have found that double-positive CCR2 + CX3CR1 + monocytes are preferentially recruited to the kidneys where they acquire macrophage markers during glomerulonephritis (Mysore et al. 2022). Monocyte recruitment to the kidneys is compromised by the reduction of the proportion of TrMac macrophages (Richoz et al. 2022). Moreover, TrMacs are crucial in the resolution and improvement of local inflammation. In comparison to healthy controls, renal biopsies of LN patients show upregulation of genes encoding anti-inflammatory mediators (Granulin [GRN], Thymosin beta-4, X-linked [TMSB4X], cAMP response element-binding protein 5 [CREB5]), and CX3CR1 and interferon-stimulated genes). Conversely, genes encoding pro-inflammatory mediators (Wingless-type MMTV integration site family, member 5 A [WNT5A], Arachidonate 15-lipoxygenase, type B [ALOX15B]) are downregulated in TrMacs. This implies that TrMacs may be drawn to local inflammatory regions, both exacerbating and attempting to resolve the inflammation resulting from MoMacs (Arazi et al. 2019). Therefore, TrMacs in LN demonstrate a dual function and may serve as a balancing role. MoMacs are derived from monocytes and have a high expression of CD11b and a low expression of F4/80. MoMacs reveal a higher level of FcγR response genes in LN, significant internalization and recycling of immune complexes (ICs), and the presentation of IC-modulated antigens (Mysore et al. 2022). During pathological conditions, macrophages can switch their energy production state from mitochondrial respiration to glycolysis. In contrast to MoMacs, TrMacs have increased sensitivity to IC immune challenge and are more effective in eliminating immune complexes, as well as higher glycolytic and glucose-uptake abilities under pathological conditions (Liu et al. 2020a). The consistent association between glycolytic capacity and the level of response to immune challenge suggests a close relationship between glycolysis and the function of macrophages.

Based on their cell marker profiles and cytokine expression, macrophages can be classified into M1 and M2 phenotypes. MoMacs can differentiate easily into either M1 or M2 cells upon cytokine stimulation. Conversely, TrMacs do not produce arginase or nitric oxide when stimulated by cytokines but acquire a mixture of pro- and anti-inflammatory functional phenotypes during nephritis. The infiltration of these macrophages with mixed inflammatory phenotypes may be related to sustained immune complex-mediated injury and exposure to circulating inflammatory mediators, which could indicate a failure to resolve inflammation and potentially result in excessive tissue remodeling during chronic inflammation. This type of macrophage population is also observed in LN kidneys, along with the classical M1 and M2 cells (Sahu et al. 2014). The pro-inflammatory type of macrophages, classically activated M1 (Inducible nitric oxide synthase [iNOS+]/CD68+), can be induced by exposure to IFNγ, lipopolysaccharide (LPS), TNFα, or granulocyte-macrophage colony-stimulating factor (GM-CSF). These macrophages express markers such as iNOS and the pro-inflammatory cytokines IL-1β, TNFα, and IL-6, which enhance resistance to infection.

**Fig. 1:**
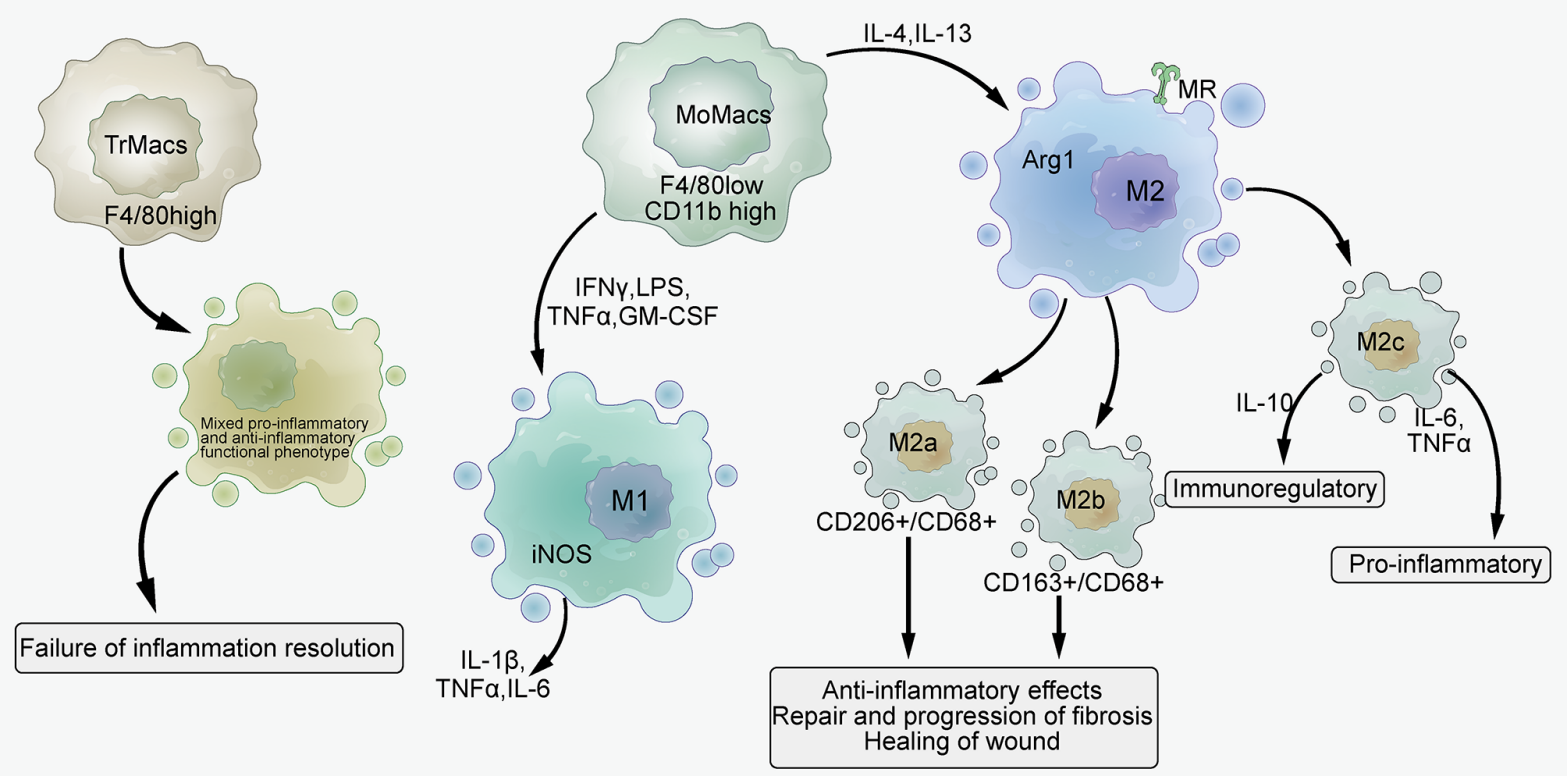
Image showing macrophage polarization. Tissue-resident macrophages (TrMacs) acquire a mixed pro-inflammatory and anti-inflammatory functional phenotype upon stimulation by cytokines, reflecting the failure of inflammation resolution. Monocyte-derived macrophages (MoMacs) polarize into M1 macrophages upon stimulation by IFNγ, LPS, TNFα, or GM-CSF, and responsible to secrete IL-1β, TNFα, and IL-6. They polarize into M2 cells by IL-4 or IL-13. M2 can be further subdivided into M2a (CD206+ / CD68+), M2b, and M2c (CD163+ / CD68+)-like macrophages. Among them, M2a and M2c have anti-inflammatory effects and are involved in fibrotic repair and progression, as well as wound healing, while M2b cells showed immunomodulatory characteristics *via* the expression of IL-10. MR: Mannose receptor

**Fig. 2:**
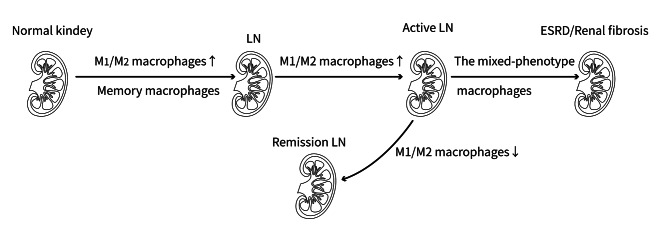
Association between macrophage polarization and disease progression. LN: Lupus nephritis; ESRD: End-stage renal disease

Conversely, M2 (F4/80Cd11c + − MR) macrophages that are stimulated by IL-4 or IL-13 show a decreased level of pro-inflammatory markers but higher levels of arginase-1, mannose receptor (MR), and IL-10. These macrophages are implicated in the regulation of inflammatory responses and the promotion of tissue repair (Lee et al. 2011). Further, M2 macrophages can be subdivided into M2a (CD206+/CD68+) cells that are induced by Th2 cell cytokines, M2b-like macrophages induced by immune complexes and LPS, and M2c (CD163+/CD68+) cells induced by glucocorticoids. Among them, M2a and M2c cells have anti-inflammatory effects and participate in fibrotic repair and progression, as well as wound healing (Sahu et al. 2014). M2b cells express IL-10 and have immunoregulatory features, however, they also secrete the pro-inflammatory cytokines TNFα, IL-6, and IL-1 (Lee et al. 2020) (Figs. 1 and 2). Studies have found that polarization of macrophages toward M2a differentiation can reduce renal injury and prolong survival, thus improving LN (Liang et al. 2021). M2c-like macrophages are recognized as contributing to the formation of active crescentic glomerulonephritis in LN (Li et al. 2017b). The number of M2c-like macrophages is significantly correlated with the renal pathology Austin activity score in all lupus kidney types classified based on the International Society of Nephrology/Renal Pathology Society (ISN/RPS) (Olmes et al. 2016). Thus, M1 macrophages can promote the development and progression of LN, whereas M2a macrophages have a positive impact on LN. However, more study is needed to determine the functions of M2c and M2b macrophages in LN.

Further study has shown that the presence of innate immune memory is significant in the progression of SLE (Chen et al. 2022a). Metabolites can promote the differentiation of MoMacs into memory macrophages by regulating epigenetic changes. These metabolites, which include ATP, NAD+, acetyl coenzyme A, and S-adenosylmethionine (SAM), have been discovered to regulate enzymes responsible for nucleosome distribution, DNA methylation, and histone modifications, which promote the development of immune memory (Kloc et al. 2022). Chen et al. observed that susceptible MRL/lpr mice with lupus revealed differences in various metabolic pathways in MoMacs, such as glycolysis, oxidative phosphorylation (OXPHOS), glutamine metabolism, fatty acid, and cholesterol synthesis, after the second stimulation. Among them, a high glycolytic metabolic rate forms the metabolic basis of innate immune memory by controlling histone methylation and acetylation. Innate immune memory enhances the ability of innate immune cells to respond to secondary stimuli and release proinflammatory factors, accelerating LN formation (Chen et al. 2022a).

## Relationship between Macrophage phenotype and metabolism

### Glucose metabolism

#### Glycolysis

Glucose is initially transported into cells *via* glucose transporters and then phosphorylated by hexokinase to produce glucose-6-phosphate (G6P) in hypoxic conditions. The conversion of G6P to pyruvate occurs through a series of enzymatic reactions, resulting in the production of small amounts of ATP. This process is known as glycolysis (Viola et al. 2019). Recent studies have found that when macrophages are stimulated by IgG immune complexes (IgG IC) alone, the IgG IC can crosslink with Fcγ receptors and induce glycolysis in an mTOR-hypoxia-inducible factor-1α (HIF-1α)-dependent manner (Jing et al. 2020). In MoMacs from lupus-prone mice, overexpression of glycolysis-associated genes has also been observed (Noe et al. 2021). Moreover, HIF1α enhances the expression of both pyruvate dehydrogenase kinases 1 (PDK1) and lactate dehydrogenase A (LDHA) (Kim et al. 2006). PDK1 inhibits the activity of pyruvate dehydrogenase, preventing the entry of pyruvate into the TCA cycle and promoting its conversion to lactate (Jung et al. 2019). Increased glycolysis and elevated lactate secretion can promote macrophage polarization in the M1 type (Wang et al. 2017). Patients with SLE who are also diagnosed with macrophage activation syndrome have higher levels of lactate dehydrogenase in macrophages and more severe renal impairment (Liu et al. 2018). This suggests that LDHA may be upregulated in the macrophages of patients with LN, resulting in higher lactate production and M1 macrophage polarization. The activation of M1 macrophages by IgG IC has been reduced by the inhibition of either glycolysis or HIF1α, resulting in an improvement in LN (Jing et al. 2020).

**Fig. 3:**
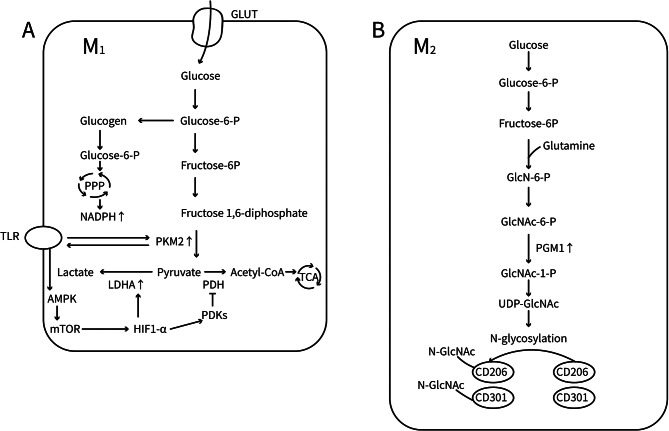
Diagram of glycometabolism in Macrophages. **(A)** Glycolysis and pentose phosphate pathway in M1 macrophages. **(B)** Amino sugar and nucleotide sugar metabolism in M2 macrophages. GLUT: Glucose transporter; PKM2: Pyruvate kinase muscle type 2; LDHA: Lactate dehydrogenase A; PDKs: Pyruvate dehydrogenase kinases; PDH: Pyruvate dehydrogenase; GlcN-6-P: Glucosamine-6-phosphate; GlcNAc-6-P: N-Acetylglucosamine-6-phosphate; GlcNAc-1-P: N-Acetylglucosamine-1-phosphate; UDP-GlcNAc: Uridine diphosphate N-acetylglucosamine

Moreover, increased levels of glucose transporters in macrophages can result in more efficient glucose uptake and glycolysis, as well as the promotion of M1 polarization in macrophages (Freemerman et al. 2014). Empagliflozin, an inhibitor of sodium-glucose co-transporter 2 (SGLT2), can reduce glucose uptake and decrease glycolysis levels, thereby reducing the levels of M1 macrophages, inducing proliferation of M2 macrophages, and alleviating tissue inflammation (Xu et al. 2019). A clinical study found that the treatment of five patients with LN with Empagliflozin had additional protective effects on the kidneys (Morales and Galindo 2022). Further, the conversion of phosphoenolpyruvate to pyruvate during glycolysis is facilitated by pyruvate kinase M2 (PKM2). The activation of the TLR4, TLR7, and TLR9 pathways in macrophages can elevate PKM2 levels. In turn, the activation of the TLR4, TLR7, and TLR9 pathways is facilitated by the overexpression of PKM2, resulting in a vicious cycle (Fig. 3A). The activation of the TLR4, TLR7, and TLR9 pathways can be substantially inhibited by the addition of PKM2 inhibitors or by disruption of PKM2 expression. Alternatively, the upregulation of PKM2 can be induced by increasing the activation of the TLR pathways in macrophages, thereby stimulating and sustaining its pro-inflammatory effects. Moreover, in vivo studies showed that treatment with PKM2 inhibitors significantly reduced the glomerular deposition of IgG and IgM in lupus mice induced by imiquimod (a TLR7 agonist) thus protecting the mice against lupus progression (Zhang et al. 2021). These results indicate that it may be possible to treat LN by inhibiting macrophage M1 polarization *via* direct suppression of glycolysis-related enzymes and proteins or by interfering with factors that enhance glycolysis levels (Jing et al. 2020).

#### Pentose phosphate pathway (PPP)

This pathway produces a significant amount of reduced coenzyme II (NADPH) through glucose metabolism. This stabilizes the redox homeostasis within M1 macrophages and enhances the stability of their metabolic and biosynthetic activities. According to a study that employed ^13^C tracing, the G6P in the PPP pathway is not directly produced from glycolysis, but rather from glycogen catabolism. The G6P that is produced from the phosphorylation of glucose by hexokinase is mainly used for glycogen synthesis. The glycogen is in turn metabolized to regenerate G6P, which is then included in the PPP pathway (Fig. 3A). This can be associated with two potential causes: the unique compartmentalization of the enzymes involved and the capacity of glycogen metabolism to activate the UDPG-P2Y14 signaling pathway, which controls the expression of inflammatory genes in macrophages and results in M1 polarization. Disruption of either the PPP or glycogen catabolism leads to downregulation of iNOS, TNF, IL-6, and IL-1β expression in IFN-γ/LPS-treated macrophages, inhibiting M1 polarization (Ma et al. 2020). Clinical studies have also reported significant increases in PPP levels in monocyte/macrophages in patients with lupus (Grayson et al. 2018). In lupus-affected kidneys, CD68 macrophages are particularly closely associated with elevated PPP levels, which are the primary cause of decreased glomerular filtration rates (Xu et al. 2022a). These results suggest that the PPP pathway in macrophages plays a crucial role in the pathogenesis of LN and may be involved in the maintenance of M1 polarization.

#### Amino sugar and nucleotide sugar metabolism

Jha et al. discovered two previously unreported M2-specific metabolic modules, namely, amino sugar and nucleotide sugar metabolism, characterized by increased levels of UDP-GlcNAc, UDP-glucose, and UDP-glucuronate, as well as transcriptional upregulation of enzymes (e.g., Ectonucleotide Pyrophosphatase/Phosphodiesterase 1 [ENPP1] and Phosphoglucomutase 1 [PGM1]) involved in the production of these intermediates. Similar to the known hexosamine biosynthetic pathway, glucose and glutamine serve as the primary sources of carbon and nitrogen, respectively, in UDP-GlcNAc. UDP-GlcNAc is a critical intermediate in the process of signal transduction and metabolism, and it is involved in the O- and N-glycosylation of serine and threonine residues in proteins. Transcriptional upregulation of various steps in the N-glycan pathway has been observed in M2 macrophages. Further, the highly glycosylated lectin/mannose receptor (MR, also named CD206) has been reported as one of the most common indicators of M2 macrophage polarization (Jha et al. 2015) (Fig. 3B). The lectin/mannose receptor can be exploited to convert M2b macrophages to an anti-inflammatory phenotype, thereby enhancing LN (Cai et al. 2013). It has been demonstrated that the N-glycosylation inhibitor tunicamycin substantially reduces the expression of the classical markers of M2 activation, including Resistin-like molecule alpha (Relmα), CD206, and CD301, while slightly affecting M1 polarization. Similarly, the hexosamine pathway is inhibited by glucosamine, which leads to M2-specific defects (Jha et al. 2015). The results indicate that increasing the activity of the UDP-GlcNAc synthesis pathway is important in promoting M2 macrophage polarization and suggests that targeting this pathway could potentially be used as a treatment option for LN.

### Lipid metabolism

#### Fatty acids

**Fig. 4:**
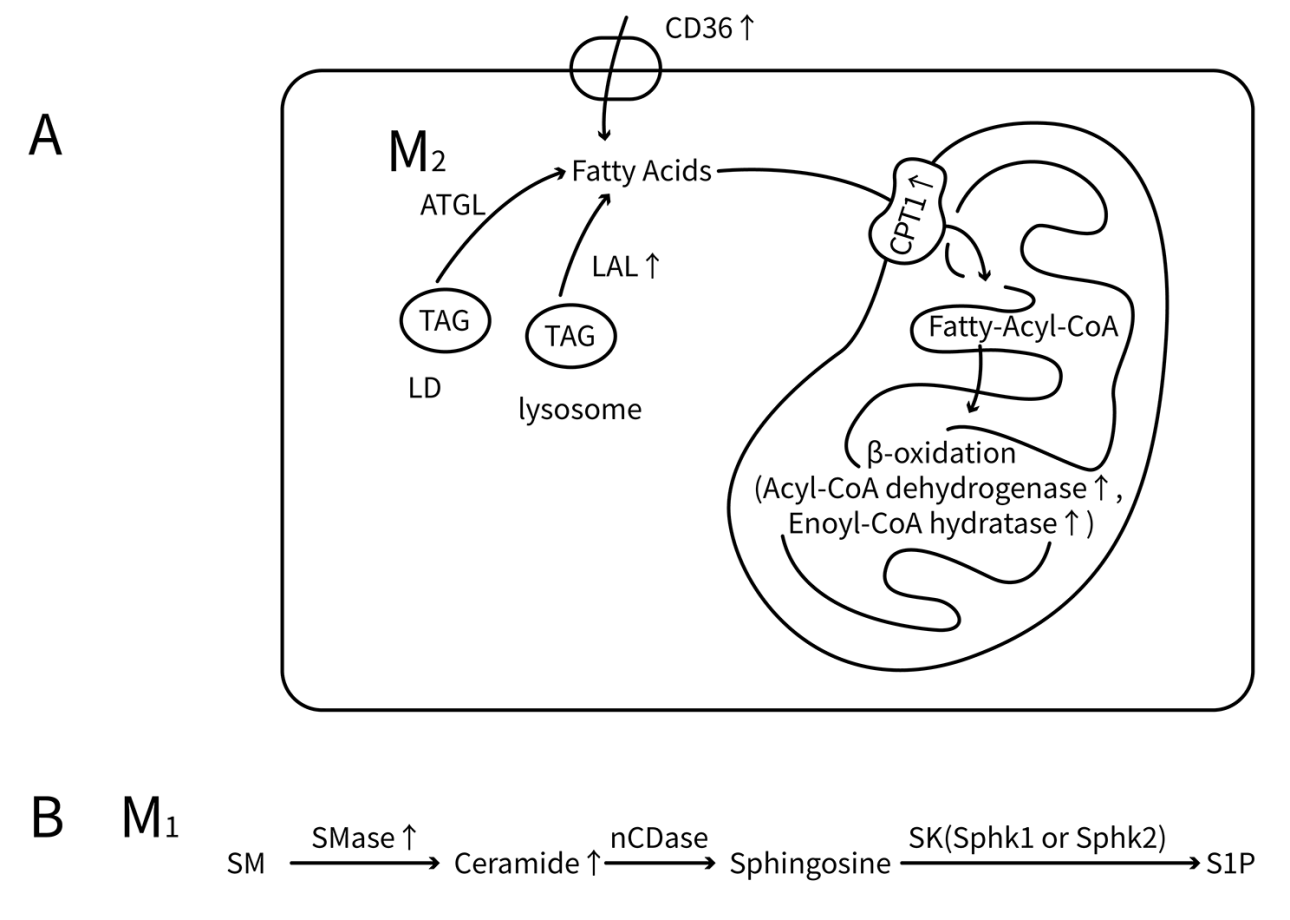
Diagram of lipid metabolism in Macrophages. **(A)** Fatty acid metabolism in M2 macrophages. **(B)** Sphingomyelin metabolism in M1 macrophages. LD: Lipid Droplet; TAG: Triacylglycerol; ATGL: Adipose Triglyceride Lipase; LAL: Lipoprotein Lipase; CPT1: Carnitine Palmitoyltransferase 1; SM: Sphingomyelin; SMase: Sphingomyelinase; nCDase: neutral ceramidase; SK: Sphingosine Kinase; Sphk1: Sphingosine Kinase 1; Sphk2: Sphingosine Kinase 2; S1P: Sphingosine-1-Phosphate.

Fatty acid oxidation (FAO) is a critical factor in the M2 polarization. Under the action of IL-4, STAT6 and peroxisome proliferator-activated receptor gamma (PPARγ) coactivator 1-beta (PGC-1β) can induce FAO in macrophages, together with mitochondrial biogenesis, leading to polarization toward the M2 phenotype. Overexpression of PGC1β enhances M2 macrophage polarization when stimulated with IFN-γ and LPS (Vats et al. 2006). In M2-polarized macrophages, increased expression of enzymes involved in FAO (acyl-CoA dehydrogenase and enoyl-CoA hydratase) and the key FAO regulatory factor PPARγ are observed in the mitochondria. Furthermore, the upregulation of proteins involved in fatty acid uptake and transport, including Lipoprotein Lipase (LPL), CD36, and Carnitine Palmitoyltransferase 1 (CPT1), in M2 macrophages is indicative of an improved fatty acid uptake by the cells (Fig. 4A). Essentially, macrophages acquire fatty acids through three pathways. The first of these is through the breakdown of triglycerides in lipid droplets (LDs), which is initiated by adipose triglyceride lipase (ATGL), second, through uptake of exogenous triacylglycerol through endocytosis mediated by the CD36 receptor, and third, through lysosomal acid lipase (LAL)-mediated lysosomal lipolysis. LAL is increased during M2 macrophage polarization, thus promoting lipolysis (Martinez et al. 2006). The expression of FAO-associated genes is regulated by the fatty acids produced by lipolysis, which function as ligands for nuclear receptors (such as PPARs). Lipolysis inhibition can effectively prevent the polarization of M2 macrophages (Huang et al. 2014). Previous studies have found that drugs targeting and activating PPAR receptors, such as pioglitazone and rosiglitazone, can promote monocyte differentiation into M2 macrophages, leading to improvements in LN (Aprahamian et al. 2009; Sun et al. 2022). Therefore, the polarization of M2 macrophages is influenced by improved FAO and increased fatty acid uptake and the above metabolic pathways may be targeted to enhance LN.

#### Phosphatidylcholine

Ceramide is produced by the hydrolysis of phospholipids by enzymes from the sphingomyelinase family (SMases), which is then converted to sphingosine by neutral ceramidase (nCDase). Sphingosine is phosphorylated by sphingosine kinases (Sphk1 or Sphk2) resulting in sphingosine-1-phosphate (S1P) (Pérez-Villavicencio et al. 2022) (Fig. 4B). Studies have found that sphingosine levels are significantly reduced in patients with LN, with a sensitivity of 87.50% and a specificity of 32.14% for identifying LN (Li et al. 2017a). Moreover, compared to healthy controls and SLE patients without kidney involvement, LN patients exhibited significantly elevated levels of lactosylceramide (Lact-Cer) in urine, as well as C16:0, C18:0, C20:0, and C24:1 ceramide levels and S1P in serum and plasma. This suggests that these ceramides and S1P aiso have some value in differentiating kidney damage in SLE patients. Dysregulation of sphingolipid metabolism may be associated with the pathogenesis of lupus nephritis (Harden and Hammad 2020; Nowling et al. 2015). In vitro experiments have shown that LPS can increase the levels of iNOS and SMase, as well as C16:0, C18:0, C20:0, and C24:1 ceramides, in macrophages, suggesting a role for SMase and ceramides in macrophage polarization towards the M1 phenotype (Józefowski et al. 2010). Sphk2 is a key enzyme involved in S1P production, and its deficiency or inhibition can prevent LN by inhibiting S1P production and promoting the polarization of macrophages towards the M2 phenotype (Ghosh et al. 2018; Snider et al. 2013). These data suggest that the increased catabolism of sphingolipids favor M1 polarization of macrophages, and the changes in sphingolipid metabolites can be used for the differential diagnosis of LN. Targeted inhibition of sphingolipid metabolism may improve LN by regulating macrophage polarization toward the M2 phenotype.

### Amino acid metabolism

#### Arginine

**Fig. 5:**
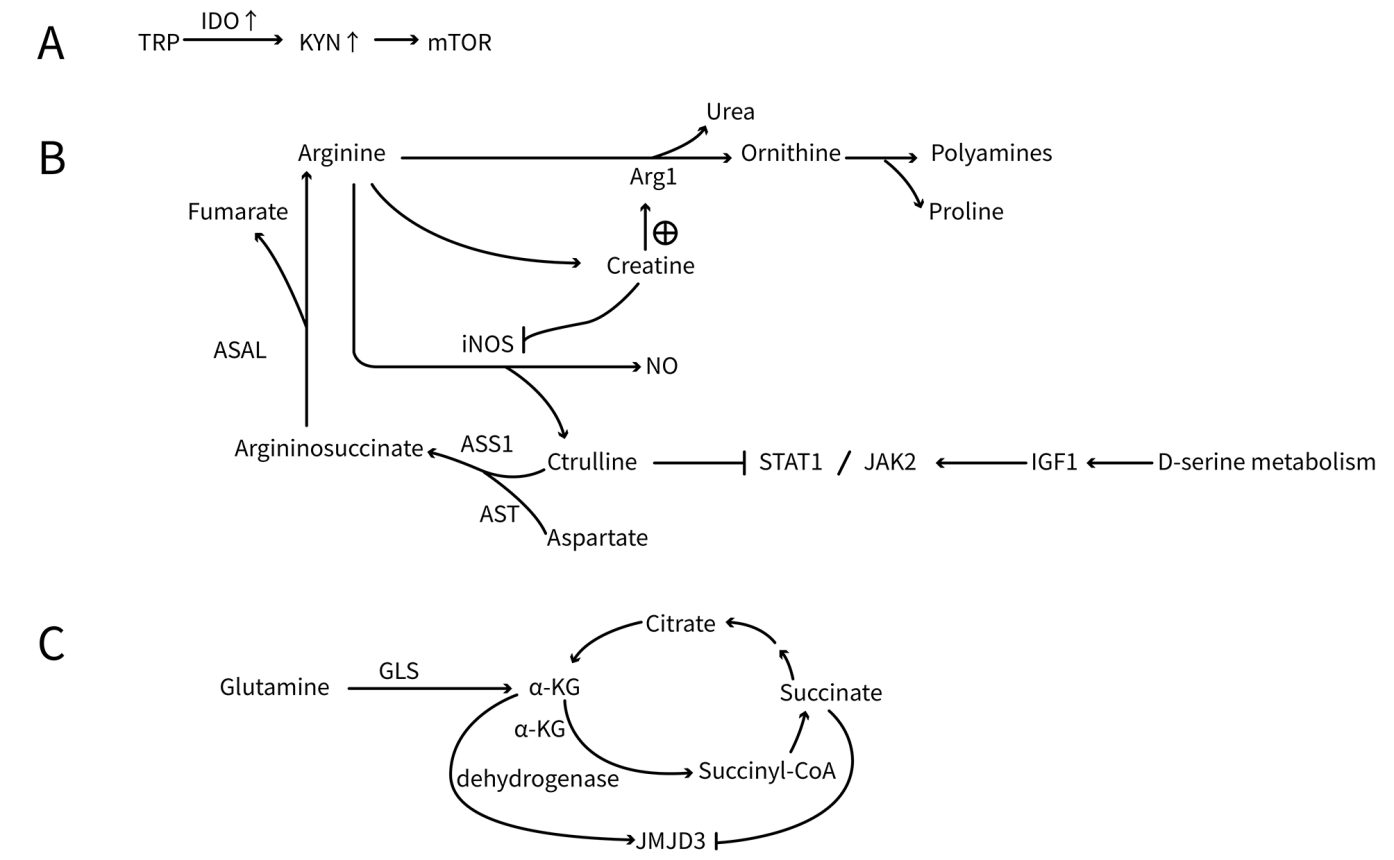
Diagram of amino Acid Metabolism in Macrophages. **(A)** Tryptophan metabolism in M1 macrophages. **(B)** Arginine and serine metabolism in macrophages. **(C)** Glutamine metabolism in macrophages. TRP: Tryptophan; KYN: Kynurenine; Arg1: Arginine; iNOS: Inducible nitric oxide synthase; ASS1: Argininosuccinate synthase 1; AST: Aspartate aminotransferase; IGF1: Insulin-like growth factor 1; GLS: Glutaminase

In macrophages, two L-arginine metabolic pathways mediated by iNOS and Arginase-1 (Arg1) play crucial roles in the M1 and M2 polarization processes, respectively (Ji et al. 2019). In M1 macrophages, the metabolic disruption of isocitrate dehydrogenase (IDH), the enzyme that converts isocitrate to α-ketoglutarate (α-kG), leads to the breakdown of the TCA cycle (Santarsiero et al. 2018). However, the diversion of the aspartate-argininosuccinate shunt can compensate for this disruption. Argininosuccinate can generate fumarate and arginine under the action of argininosuccinate lyase. Fumarate supplements the downstream cycle and provides substrates for citrate synthase, increasing the itaconate flux and resulting in the maintenance of the M1 phenotype. Arginine is metabolized by iNOS to produce NO and citrulline, and citrulline can be reused through the arginine-nitric oxide cycle, promoting the synthesis of NO (Jha et al. 2015). NO competes with oxygen, inhibits complex II of the electron transport chain (succinate dehydrogenase), and reduces the conversion of succinate to fumarate in the TCA cycle, thus generating the second breakpoint described above, and promoting macrophage polarization toward M1 (Stadler et al. 1991). Furthermore, citrulline can also act as an anti-inflammatory signal by inhibiting JAK2/STAT1 signaling to prevent M1 macrophage activation. Therefore, the timely consumption of citrulline through the conversion of citrulline to argininosuccinate by argininosuccinate synthase 1 (ASS1) in the urea cycle and arginine-nitric oxide cycle is important for the maintenance of M1 macrophages. Moreover, upregulation of ASS1 has also been observed in M1 macrophages (Fig. 5B). The provision of exogenous citrulline to cells can inhibit the mRNA levels of inflammatory genes in IFN-γ-stimulated macrophages, thereby inhibiting M1 macrophage polarization (Mao et al. 2022). In addition, inhibition of aspartate aminotransferase, a key enzyme that converts aspartate to argininosuccinate, can suppress the production of NO and IL-6 by M1 macrophages, while promoting mitochondrial respiration, which is beneficial for the polarization of macrophages towards the M2 phenotype (Jha et al. 2015). Therefore, it can be seen that the inhibition of aspartate aminotransferase, iNOS, and ASS1, or the provision of exogenous citrulline, can reduce M1 macrophage activation. Conversely, in M2 macrophages, the expression of Arg1 is upregulated, which hydrolyzes L-arginine into L-ornithine and urea, limiting the synthesis of NO. L-ornithine further participates in the synthesis of polyamines and proline, both of which are important for cell proliferation and tissue repair (Salminen 2021). Another metabolic pathway associated with L-arginine, creatine metabolism, has also been reported to play a role in macrophages. From a metabolic perspective, creatine can promote macrophage polarization toward the M2 phenotype by inhibiting iNOS and enhancing the expression of Arg1, forming a tight arginine immune metabolic network (Ji et al. 2019). Therefore, upregulation of the creatine metabolic pathway associated with L-arginine and the metabolic pathway mediated by Arg1, together with downregulation of the metabolic pathway mediated by iNOS, may serve as therapeutic targets for LN. Njoku et al. found that iNOS inhibitors can reduce urinary biomarkers of systemic oxidative stress in mice with proliferative LN, further supporting this point (Njoku et al. 2005).

#### Tryptophan

The autoimmune activation of lupus-prone mice may be significantly influenced by the abnormal activation of tryptophan metabolism (Choi et al. 2020). Tryptophan is predominantly metabolized via the kynurenine (KYN) pathway, where it is converted to kynurenine by indoleamine 2,3-dioxygenase (IDO). Both the total IDO enzyme activity and the IDO protein were substantially elevated in the lupus-prone B6.Nba2 model in comparison to normal B6 mice (Davison et al. 2019). Clinical observations have demonstrated that patients with SLE show elevated levels of Kyn and reduced levels of Trp in the blood. An increased Kyn/Trp ratio in the blood is consistent with increased IDO activity and was found to be associated with disease activity (Åkesson et al. 2018; Badawy and Guillemin 2019). The novel metabolic checkpoint in lupus pathogenesis may be the result of the activation of mTOR by elevated levels of KYN (Perl et al. 2015) (Fig. 5A). It is essential to activate the TLR/AMPK/mTORC1 signaling pathway to induce the M1-like metabolic phenotype and IL-12 secretion. IDO expression has also been observed to be high in M1 macrophages (Xu et al. 2022a). These results indicate that the abnormal metabolism of tryptophan may enhance the polarization of M1 macrophages and contribute to the LN development. Davison et al. discovered that patients with SLE with renal involvement improved from appropriate IDO inhibition (Davison et al. 2019). Thus, it is imperative to conduct research that focuses on tryptophan metabolism to modulate macrophage polarization and enhance LN.

#### Serine

Serine metabolism is related to the functionality of immune cells. Inhibiting serine metabolism, either by inhibiting the activity of phosphoglycerate dehydrogenase, a key enzyme in the serine biosynthetic pathway, or by exogenous serine and glycine restriction, has been found to effectively suppress M1 macrophage polarization both in vitro and in vivo, while enhancing the polarization of M2 macrophages (Shan et al. 2022). Mechanistically, a deficiency in serine metabolism reduces the levels of S-adenosylmethionine-dependent histone H3 lysine 27 trimethylation (H3K27me3), thereby increasing the expression of insulin-like growth factor 1 (IGF1). Subsequently, IGF1 activates the p38-dependent JAK/STAT1 axis, promoting M1 polarization and inhibiting STAT6-mediated M2 activation (Shan et al. 2022) (Fig. 5B). Further, serine can be converted to glycine, which regulates the expression of the microRNA miR-301a, thereby promoting macrophage polarization toward the M2 phenotype (Gan et al. 2021). In LN, the disease activity is closely correlated with reductions in D-serine (Okushima et al. 2021), suggesting that appropriate upregulation of serine metabolism may serve as a therapeutic strategy for LN.

#### Glutamine

The metabolism of glutamine-glutamate plays a synergistic role in regulating macrophage differentiation, rather than having a direct effect. M2 polarization triggers the catabolism of glutamine and UDP-GlcNAc-related modules (Jha et al. 2015). In M2 macrophages, glutamine is the source of approximately one-third of the TCA metabolic carbon, compared to M1 cells (20%). When ^15^N-labeled glutamine was added, it was observed that more than half of the nitrogen in UDP-GlcNAc was derived from glutamine within 4 h. The breakdown product of glutamine, αKG, not only enters the TCA cycle to replenish intermediates but also serves as a co-stimulatory factor for JMJD3, promoting M2 activation through JMJD3-dependent metabolic and epigenetic reprogramming. Moreover, αKG reduces the pro-inflammatory response of M1 macrophages by inhibiting the nuclear factor-κB (NF-κB) pathway *via* prolyl hydroxylase (PHD)-dependent prolyl hydroxylation of protein kinase IKKβ (Liu et al. 2017). Furthermore, αKG can be converted to succinate through the αKG dehydrogenase complex and succinyl-CoA, and succinate inhibits JMJD3 (Fig. 5C). Studies have shown changes in the activity of αKG dehydrogenase between M1 and M2 macrophages. Suppression of αKG dehydrogenase results in an increase in the αKG/succinate ratio, which in turn promotes the expression of M2-specific marker genes and Arg1 activity (Liu et al. 2017; Tannahill et al. 2013). The deprivation of glutamine leads to a substantial reduction in the levels of M2-specific marker genes (Arg1, Interferon Regulatory Factor 4 [IRF4], Kruppel-Like Factor 4 [KLF4], Chemokine [C-C motif] Ligand 22 [CCL22], and IL4), as well as reduced transcription of TCA cycle components, thereby decreasing the cycle’s activity, while increasing the expression of M1-sensitive marker genes, including IL1β, TNF, IL6, and IL12, suggesting a close association between glutamine metabolism and macrophage polarization (Jha et al. 2015; Liu et al. 2017). Therefore, enhancing the catabolism of glutamine and inhibiting αKG dehydrogenase can promote M2 polarization of macrophages.

#### Amino acid transport proteins

Studies have demonstrated that the activity of amino acid transport proteins is essential for macrophage activation ((Kobayashi et al. 2021). These proteins can function as guardians, enabling macrophages to acquire a metabolic phenotype that favors M1 polarization. The expression of the amino acid transport protein SLC7A5 is significantly induced within 24 h under conditions that favor M1 polarization. Uptake of amino acids, especially leucine, mediated by SLC7A5 can enhance mTORC1-mediated glycolysis and promote macrophage polarization toward M1 (Yoon et al. 2018). Further, studies have found that a lysosomal-resident amino acid/oligopeptide transporter, SLC15A4, expressed in immune cells, plays a key role in the pathogenesis of lupus. The steady-state expression of SLC15A4 in these innate immune cells safeguards their ability to adapt their metabolic function to the proinflammatory state by connecting glycolysis and the TCA cycle while restricting the levels of glutaminolysis. Loss of SLC15A4 can lead to insufficient biotransformation of pyruvate for the TCA cycle, while increasing the contribution of glutaminolysis to the cycle (Kobayashi et al. 2021). A Toll-like receptor adaptor protein (TASL) was discovered to interact with SLC15A4 in lysosomes when human monocyte-derived macrophages were treated with IFN-β. This interaction connects the intralysosomal TLR with the IRF4 transcription factor and contributes to the development of SLE (Heinz et al. 2020). These results indicate that the steady-state expression and overexpression of the amino acid transport proteins SLC7A5 and SLC15A4 are favorable for macrophage M1 polarization. Therefore, the downregulation and inhibition of these two amino acid transport proteins may be effective intervention targets for both SLE and LN (Baccala et al. 2013).

### TCA pathway

**Fig. 6:**
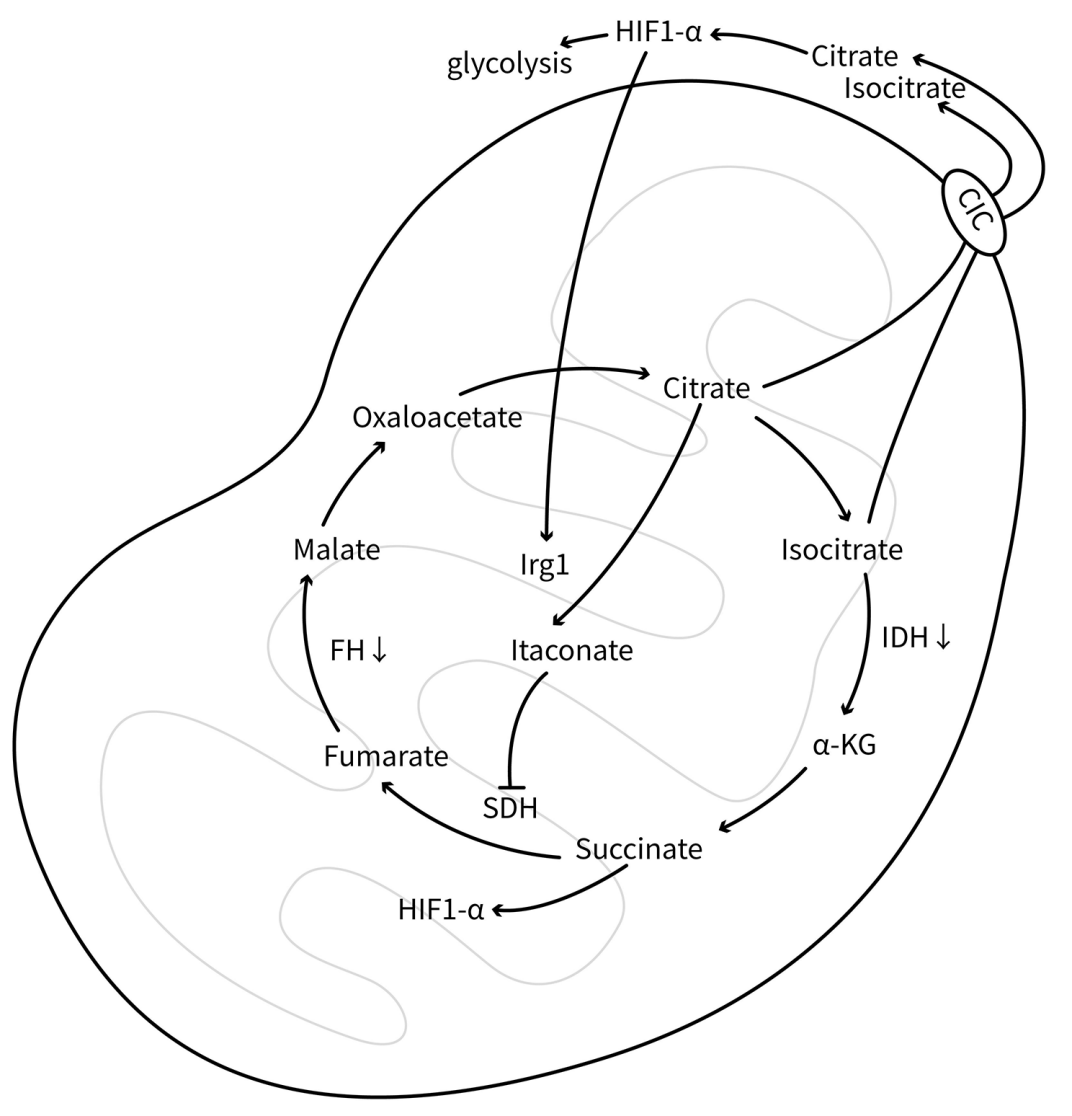
Diagram of TCA in M1 Macrophages. FH: Fumarate Hydratase; SDH: Succinate dehydrogenase; Irg1: Interferon regulatory factor 1; IDH: Isocitrate dehydrogenase. CIC: Citrate carrier

A complete and functional TCA cycle is essential for the maintenance of the differentiation into the M2 phenotype, as M2 macrophages depend on OXPHOS for ATP generation (Feng et al. 2023; Koelwyn et al. 2018; Viola et al. 2019). Inhibition of fumarate hydratase (FH), one of the enzymes in the TCA cycle, results in the accumulation of fumarate in macrophages, disrupting the TCA cycle and suppressing M2 polarization. Clinical studies have shown that FH expression in whole-blood samples of patients with SLE is significantly decreased, suggesting that M2 macrophage polarization may be suppressed in SLE patients (Hooftman et al. 2023). Citrate/isocitrate is one of the substances in the TCA cycle that can be exported from mitochondria *via* the mitochondrial citrate carrier (CIC) to modulate HIF-1α-dependent genes and stimulate glycolysis. Concurrently, HIF-1α can stimulate the transcription of immune-responsive gene 1 (Irg1), which results in the conversion of citrate to itaconate (Li et al. 2022). Accumulation of itaconate inhibits succinate dehydrogenase, resulting in succinate accumulation, which in turn upregulates HIF-1α and activates the transcription of glycolysis-associated genes to maintain the M1 phenotype (Puchalska et al. 2018; Viola et al. 2019) (Fig. 6). The suppression of CIC can suppress glycolysis, block the Irg1/itaconate pathway to ensure normal mitochondrial cycling, prevent the accumulation of citrate and itaconate, and reduce the inhibition of succinate dehydrogenase. Consequently, OXPHOS flux is improved, and the phenotypic transition of macrophages from M1 to M2 is promoted (Li et al. 2022; Puchalska et al. 2018). This may offer a better understanding of the treatment of LN.

## Targeting metabolic reprogramming to regulate macrophage polarization for alleviating LN

### Glycolysis

#### Fumarate derivatives

The active site cysteine of the glycolytic enzyme GAPDH is the primary target of endogenous fumarate salts, which can succinate proteins (Blatnik et al. 2008). Studies have found that dimethyl fumarate (DMF) and its clinically relevant metabolite monomethyl fumarate (MMF) can target GAPDH to inactivate its enzymatic activity both in vitro and in vivo (Kornberg et al. 2018). Furthermore, DMF can reduce the secretion of TNF-α and IL-6 in LPS-stimulated bone marrow-derived macrophages (BMDMs), while increasing the production of IL-10 and Arg-1 (Tang et al. 2022), suggesting that DMF may inhibit M1 polarization and promote M2 polarization of macrophages by downregulating glycolysis. Currently, DMF is approved as an oral therapeutic agent for multiple sclerosis (MS) and is suggested as a potential treatment for various inflammatory disorders (Panda et al. 2022). In Pristane-induced LN mice, Ebihara et al. discovered that DMF inhibited the progression of kidney disease. Furthermore, the study assessed the efficacy of DMF in comparison to steroids and determined that DMF was more effective than steroids in alleviating kidney disease in LN mice (Ebihara et al. 2016). Therefore, DMF may potentially replace steroids for the clinical treatment of steroid-resistant LN.

### Lipid metabolism

#### ω-3 polyunsaturated fatty acids (PUFAs)

ω-3 PUFAs are known ligands of PPARγ, a transcription factor that promotes M2 macrophage polarization by regulating the expression of FAO-associated genes. On the other hand, ω-3 PUFAs can impede NF-κB-dependent transcription of genes associated with inflammation and autoimmunity, thereby suppressing the transcriptional activation of inflammatory factors such as TNF, IL-1, and IL-6, and inhibiting M1 polarization (Pestka et al. 2021). Krill oil (KO) is a natural product rich in ω-3 PUFAs and the potent antioxidant astaxanthin. KO significantly reduces the levels of IL-1β and TNFα in human LPS-treated macrophages in a dose-dependent manner by modulating broad-spectrum signaling pathways such as those of NF-κB and NOD-like receptor signaling, thereby inhibiting M1 polarization and promoting M2 polarization (Liu et al. 2020b). A meta-analysis showed that ω-3 PUFAs can reduce SLE disease activity (Duarte-García et al. 2020), and the use of ω-3 PUFAs such as eicosapentaenoic acid (EPA) and docosahexaenoic acid (DHA) for dietary or drug intervention can improve lupus symptoms and the levels of disease biomarkers. Studies have found that DHA can inhibit IFN activation triggered by TLR4, inhibit M1 classical activation, and promote M2 activation (Favor et al. 2023; Wierenga et al. 2022). These findings indicate that exogenous administration of ω-3 PUFAs to regulate PPARγ-mediated macrophage polarization can alleviate LN.

#### Polyenylphosphatidylcholine (PPC)

PPC is a classic hepatoprotective drug extracted from soybeans and is rich in polyunsaturated fatty acids (Pan et al. 2017). In LPS-stimulated RAW264.7 macrophage and mouse BMDM models, PPC was found to interact with TLR-2 to reduce the mRNA expression of several key enzymes involved in the glycolysis and lipid synthesis pathways, while increasing the expression of key enzymes associated with lipid oxidation (Feng et al. 2020; Luo et al. 2017). This thus inhibits M1 and promotes M2 activation in macrophages. Additionally, the interaction between PPC and TLR-2 can suppress the release of pro-inflammatory cytokines (TNF-a, IL-6) and promote the production of anti-inflammatory cytokines (IL-10, TGF-β) in macrophages, thereby promoting macrophage polarization toward M2 and alleviating the symptoms of rheumatoid arthritis (Pan et al. 2017; Xu et al. 2022b). Considering that LN is an immune-related disease similar to rheumatoid arthritis, PPC may be potentially used for the future treatment of LN.

#### Sinomenine (SIN)

The occurrence of LN depends on the production of autoantibodies and signaling through interferon alpha/beta receptor (IFNAR). Studies have shown that activators of Nrf2 can downregulate the expression of IFNAR, inhibit Inhibitor of nuclear factor kappa-B alpha (IκBα) phosphorylation, and translocation of NF-κB to the nucleus, thereby suppressing M1 polarization and promoting M2 polarization in macrophages (Han et al. 2020). Additionally, Nrf2 can upregulate the expression of CD36 and downregulate mitochondrial superoxide. SIN, an activator of Nrf2 derived from medicinal herbs, is primarily used in the Far East for the treatment of rheumatic diseases. It has been demonstrated to improve nephritis by modulating macrophage polarization (Qin et al. 2016; Zeng and Tong 2020). Therefore, SIN may hold significant therapeutic potential for LN.

### Amino acid metabolism

#### Taurine

Taurine can be used to improve renal function in various kidney diseases, including immune and toxin-induced chronic renal failure, diabetic nephropathy, glomerulonephritis, and acute kidney injury. It has been found that taurine supplementation can also alleviate LN (Luo et al. 2021). Its specific mechanism may be related to enhancing the balance in energy metabolism, repairing damage caused by inflammation, and inhibiting M1 polarization. It has been observed that during M1 macrophage polarization, transcription of the taurine transporter TauT/SLC6A6 is upregulated, although the intracellular levels of taurine in macrophages remain unaffected. After taurine supplementation, the high intracellular taurine levels in macrophages can disrupt methionine metabolism, leading to a deficiency in S-adenosylmethionine (SAM), thereby inhibiting protein phosphatase 2 A (PP2Ac) methylation to block PINK1-mediated mitochondrial autophagic flux, while maintaining high mitochondrial density, and ultimately preventing the metabolic switch to glycolysis required for M1 macrophage polarization (Meng et al. 2021). Therefore, in the future, taurine may be considered for the clinical treatment of LN.

#### Retinoic acid

Retinoic acid is a naturally synthesized derivative of vitamin A that has both antitumor and immunomodulatory properties. It has been used in the treatment of acute promyelocytic leukemia, psoriasis, acne, and rheumatoid arthritis. Although retinoids have not been used for treating human kidney diseases, various animal models have shown improvement in renal pathological outcomes and proteinuria after retinoid administration. Studies have found that retinoic acid treatment can induce the production of IL-10 and Arg1 in monocytes/macrophages, while inhibiting the production of TNF-α and IL-12, thereby suppressing M1 polarization and promoting macrophage polarization toward the M2 phenotype (He et al. 2021; Miyabe et al. 2015). Additionally, there have been case reports suggesting the efficacy of retinoic acid in the treatment of LN (Kinoshita et al. 2010). Therefore, it appears that retinoic acid may alleviate LN by regulating macrophage polarization.

#### Salbutamol

Salbutamol, a β2 receptor agonist, can inhibit and reprogram macrophage cellular metabolism. R-Salbutamol has been found to significantly reduce iNOS and downregulate the expression of typical M1 macrophage cytokines, including monocyte chemoattractant protein-1 (MCP-1), IL-1β, and TNF-α. It also reduces aerobic glycolysis and glycerophospholipid metabolism while enhancing mitochondrial respiration, thereby inhibiting M1 macrophage polarization (Wang et al. 2020). Current research has confirmed the use of salbutamol as an adjunctive therapy for SLE (Wulf and Ullman 2007). Therefore, further studies are needed to clarify the therapeutic effects of salbutamol in LN.

#### Metformin

Metformin can activate SIRT1 expression in macrophages, subsequently downregulating TLR4, p-NF-κB, IL-1β, and iNOS, and upregulating Arg1, and thereby inhibiting M1 macrophage polarization and promoting M2 polarization (Liu et al. 2023). Chen et al. also found that metformin can alleviate renal injury in patients with LN (Chen et al. 2022b).

#### Triptolide

Triptolide is an effective inhibitor of several inflammatory mediators (iNOS, IL-1β) and can downregulate the expression of iNOS and upregulate the mRNA expression of Arg1, thereby inhibiting M1 macrophage polarization (Luo et al. 2017). Research has found that it can prevent and improve LN (Xu and Wu 2002) (Table 1).

**Table 1:** Substances targeting macrophage polarization to improve lupus kidney

Targeted energy metabolic pathways	Substances	Targets	Macrophage Polarization	References
Glycolysis	Fumarate Derivatives	GAPDH	M2↑, M1↓	^73^
Lipid metabolism	ω-3 PUFAs	PPARγ	M2↑, M1↓	^76^
	Polyenylphosphatidylcholine	TLR-2	M2↑, M1↓	^82^
	Sinomenine	CD36	M2↑	^86,87^
Amino Acid Metabolism	Taurine	S-adenosylmethionine	M1↓	^89^
	Retinoic acid	Arg1	M2↑, M1↓	^90^
	Salbutamol	iNOS	M1↓	^93^
	Metformin	iNOS, Arg1	M2↑, M1↓	^95^
	Triptolide	iNOS, Arg1	M2↑, M1↓	^83^

## Lupus nephritis treatment regimens and its impact on macrophage polarization

All lupus patients without contraindications are advised to use hydroxychloroquine or effective antimalarial drugs, as per the 2024 Clinical Practice Guidelines for the Management of LN. For type I and II LN patients, low-dose glucocorticoids combined with another immunosuppressive agent is recommended. The initial treatment for active type III/IV LN is a combination of glucocorticoids with one of the following: mycophenolate analogs (MPAA); low-dose intravenous cyclophosphamide; belimumab combined with MPAA or low-dose intravenous cyclophosphamide; or MPAA combined with calcineurin inhibitors (cyclosporine, tacrolimus) when renal function is not severely impaired (severe impairment is defined as eGFR ≤ 45 mL/min/1.73 m²). MPAA maintenance treatment is recommended after induction therapy. The guidelines also specify that azathioprine may be used as an alternative to MPAA for patients who are intolerant to MPAA, unable to obtain MPAA, or planning for pregnancy. In 2024, the KDIGO guidelines have not established a definitive treatment recommendation for type V LN, but they have provided practical recommendations to guide clinical practice.

Hydroxychloroquine can inhibit the production of iNOS by macrophages (Perečko et al. 2014), downregulate M1-type markers, and upregulate some M2-type markers of macrophages, thereby promoting the polarization shift of macrophages from the M1 type to the M2 type (Shiratori et al. 2018; Zheng et al. 2021). Glucocorticoids can inhibit glycolysis by blocking the transcription and post-translational effects of HIF1α in macrophages, upregulate glutamine metabolism, promote the flux of the tricarboxylic acid (TCA) cycle, enhance succinate metabolism, prevent intracellular accumulation of succinate, and promote the transformation of macrophages from M1 to M2(Qu et al. 2023; Stifel et al. 2022). MPAA can significantly enhance the mRNA levels of CD36 and scavenger receptor class A member 1 (SR-A1) responsible for fatty acid uptake in M1 macrophages, and increase the expression of M2 surface markers (including CD163 and CD200R), promoting the polarization of macrophages from M1 to M2(Kannegieter et al. 2017; Voloshyna et al. 2019). Azathioprine and the calcineurin inhibitor cyclosporin A can inhibit iNOS in macrophages, suppressing M1 polarization(Moeslinger et al. 2006). Kannegieter et al. found that another calcineurin inhibitor, tacrolimus, can promote the polarization of macrophages to an M2-like phenotype(Kannegieter et al. 2017).

## Summary

Macrophages are the main infiltrating cells in LN kidneys. Different macrophage subtypes have different effects on the development and progression of LN. According to their origin, macrophages can be divided into MoMacs and TrMacs, both of which play coordinated roles in LN. In patients with LN, TrMacs are recruited to the area of inflammation, exacerbating inflammation, while also attempting to resolve the inflammation caused by MoMacs, and can also promote the recruitment of monocytes to the kidney. TrMac macrophages, when stimulated by cytokines, are unable to produce either arginase or nitrates, and acquire a mixed pro- and anti-inflammatory functional phenotype during nephritis. The infiltration of these mixed-phenotype macrophages may be a reflection of failed resolution of inflammation. On the other hand, MoMacs can easily differentiate into M1 or M2 cells under cytokine stimulation. The M1 phenotype is proinflammatory and secretes proinflammatory cytokines, while the M2 phenotype is essentially anti-inflammatory and promotes tissue repair. In addition, M2 macrophages can be further subdivided into M2a, M2b, and M2c classes based on the expression of different cytokines. Among them, M2a and M2c produce anti-inflammatory effects and participate in tissue repair, while M2b macrophages have immunoregulatory and proinflammatory characteristics. Studies have confirmed the beneficial effects of M2a-like macrophages in alleviating LN. However, excessive activation of M2a macrophages may promote fibrosis in LN, indicating that moderate M2a activation is likely to be of significant importance for LN (Anders and Ryu 2011). Furthermore, research has shown that memory macrophages can accelerate the progression of LN, and high levels of glycolysis form the metabolic basis for innate immune memory. Therefore, it can be seen that M1 macrophages and memory macrophages may participate in the occurrence and development of LN, while moderate activation of the M2a subtype could improve LN.

**Fig. 7:**
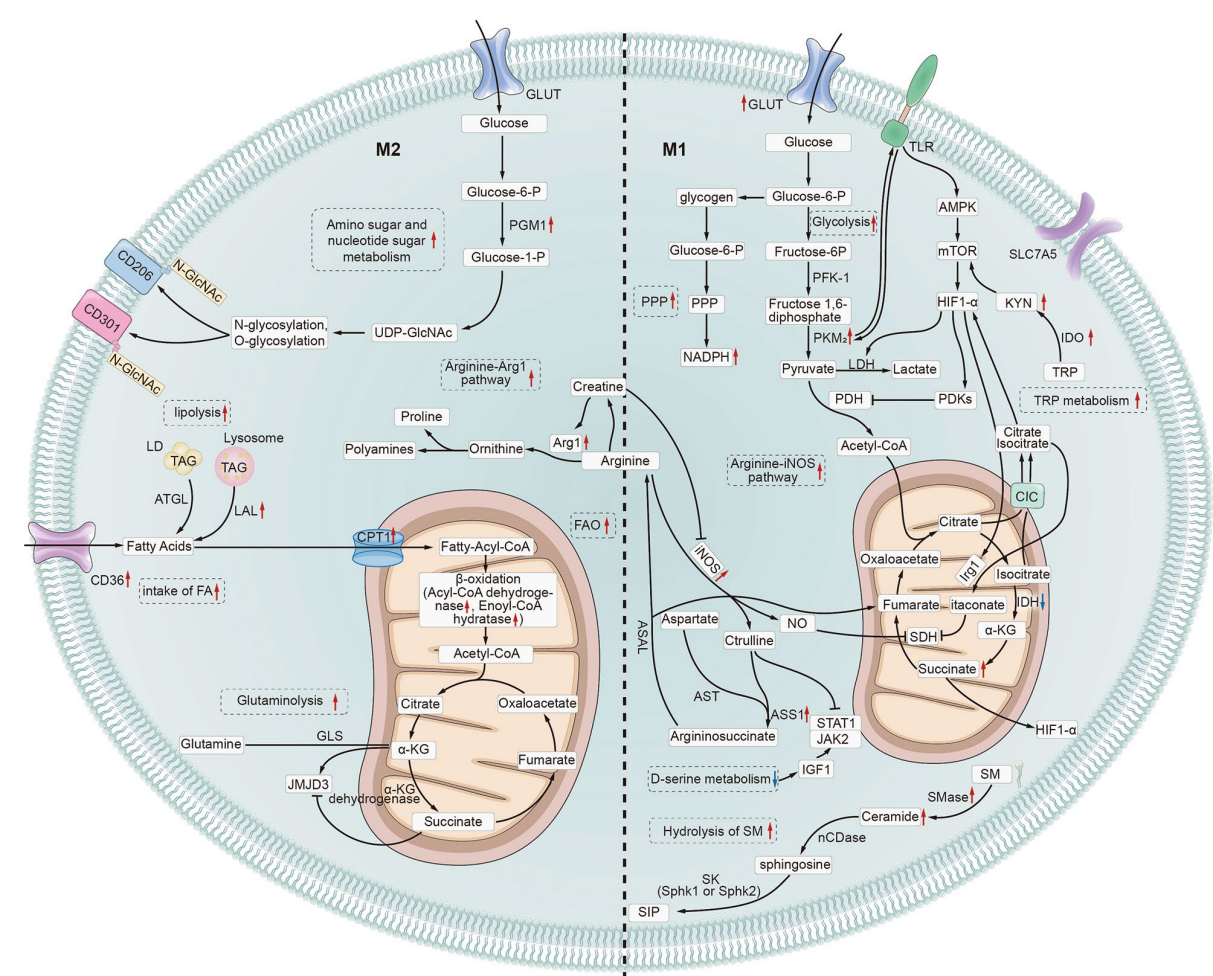
Diagram showing energy metabolism in M1 and M2 macrophages. Metabolism associated with M1 polarization of macrophages: upregulation of glycolysis and the pentose phosphate pathway, disruption of the TCA cycle, increased catabolism of sphingolipids, enhanced arginine metabolism pathway mediated by inducible NO synthase (iNOS), abnormal activation of the kynurenine (KYN) metabolism pathway of tryptophan, deficiency in serine metabolism, and steady-state expression and upregulation of amino acid transporters (SLC7A5 and SLC15A4). Metabolism associated with M2 polarization of macrophages: increased aminosugar and nucleotide sugar metabolism, upregulation of fatty acid oxidation, integrity of the TCA cycle, upregulation of the arginine metabolism pathway mediated by Arg1, and enhanced glutamine catabolism. LD: Lipid droplet; TAG: Triacylglycerol; ATGL: Adipose triglyceride lipase; FAs: Fatty acids; LAL: Lysosomal acid lipase; CPT1: Carnitine palmitoyltransferase 1; Fatty-Acyl-CoA: Fatty acyl-coenzyme A; JMJD3: Jumonji domain-containing protein 3; GLS: Glutaminase; GLUT: Glucose transporter; PGM1: Phosphoglucomutase 1; PDH: Pyruvate dehydrogenase; PFK-1: Phosphofructokinase-1; ASS1: Argininosuccinate synthase 1; ASAL: Argininosuccinate lyase; JAK2: Janus kinase 2; SDH: Succinate dehydrogenase; SM: Sphingomyelin; Smase: Sphingomyelinase; IDH: Isocitrate dehydrogenase; FH: Fumarate hydratase; CIC: Citrate carrier; nCDase: Neutral ceramidase; SK: Sphingosine kinase; TRP: Tryptophan; KYN: Kynurenine; IDO: Indoleamine 2,3-dioxygenase

Multiple studies have confirmed that energy metabolism can regulate the direction of macrophage polarization. However, most studies have focused only on M1 and M2 polarization, with less research on M2 subtypes, memory macrophages, and the third type of macrophage. These studies have identified several aspects related to M1 polarization of macrophages, specifically, upregulation of both the glycolysis and pentose phosphate pathways, disruption of TCA, increased breakdown of sphingolipids, increased iNOS-mediated arginine metabolism, aberrant activation of the tryptophan KYN metabolic pathway, reduced serine metabolism, and steady-state expression and upregulation of amino acid transporters (SLC7A5 and SLC15A4). In terms of aspects related to M2 macrophage polarization, the significant pathways include increased metabolism of amino and nucleotide sugars, upregulation of FAO, an intact TCA cycle, upregulation of the arginine metabolic pathway mediated by Arg1, and enhanced glutamine degradation (Fig. 7). Currently, several substances have been found to target these energy metabolism pathways, inhibit M1 polarization of macrophages, promote M2 polarization, and thereby improve LN. These include ω-3 PUFAs, PPC, and SIN which can upregulate the FAO pathway and promote M2 polarization, fumaric acid derivatives that can downregulate glycolysis and inhibit M1 polarization, taurine that can attenuate methionine metabolism and inhibit M1 polarization, retinoic acid, salbutamol, metformin, and celastrol for the downregulation of iNOS, upregulation of Arg1, and regulation of arginine metabolism, thus inhibiting M1 polarization and promoting M2 polarization of macrophages. It is worth mentioning that studies have suggested that long-term exposure to M2 macrophages may lead to renal fibrosis^10^. However, (Anders and Ryu 2011). found that steroid treatment also reduces renal inflammation by inducing M2 macrophage polarization, directly regulating the balance between renal inflammation and tubular epithelial healing without causing fibrosis. This indicates that M2 macrophages may play a specific role in LN through a complex mechanism. Therefore, it would be important to treat LN by appropriate intervention in macrophage pathways associated with energy metabolism to inhibit M1 polarization and promote M2 polarization through the use of suitable drugs at the right time and in a moderate manner.

Unfortunately, there is currently no research exploring the differences in energy metabolism among the M2 subtypes M2a, M2b, and M2c. The aforementioned studies only indicate that promoting M2 polarization can improve LN, without specifying which specific M2 subtype can improve LN. Therefore, future research is still required for the extensive exploration of the specific roles of the M2 subtypes in LN and changes in energy metabolism in M2 macrophages.

## Data Availability

Not applicable.
